# Dataset on physicochemical and microbial properties of raw water in four drinking water treatment plants based in South Africa

**DOI:** 10.1016/j.dib.2020.105822

**Published:** 2020-06-09

**Authors:** Vhahangwele Masindi

**Affiliations:** Magalies Water, Scientific Services, Research & Development Division, Erf 3475, Stoffberg street, Brits, 0250, Tel: 0123816602.; Department of Environmental Sciences, School of Agriculture and Environmental Sciences, University of South Africa (UNISA), P. O. Box 392, Florida, 1710, South Africa

**Keywords:** Raw water, Water treatment plants, Physicochemical parameter, Treatability index, Water quality monitoring and modelling

## Abstract

The present paper aims at determining the status of surface water quality by applying the treatability index for the raw water in four water treatment plants (WTPs), namely Vaalkop, Klipdrift, Wallmansthal, and Cullinan. These plants are based in South Africa. Sampling was conducted from July 2011 to June 2018 (7 years). The collected water samples were analysed on monthly basis over the specified period. Three Hundred and thirty six (336) water samples were collected and analysed. The Treatability Index (TI) was calculated for twenty-one physicochemical and microbial parameters, which include pH, conductivity, chloride, sodium, potassium, hardness, alkalinity, precipitation potential, turbidity, colour, *E. coli*, organic carbon, chlorophyll, nitrite, ammonia, nitrates, phosphate, iron, manganese, and sulphate. The computed TI values range from 0.1 to 1755.5 and the water quality was unsuitable for a number of defined uses. The data demonstrated high treatment demand for raw water. On that note, the surface water from the monitored places is not suitable for drinking purposes. The data and treatability index denoted the need for treatment prior consumption. The collected water quality data can be reused for future references, modelling, and trending of historic data to understand current and prospect future changes in the properties of our raw water qualities.

Specifications table

**Table:** 

Subject	Environmental Sciences			
Specific subject area	Water monitoring and quality			
Type of data	Table and Figure			
How data were acquired	Data was acquired through sampling and analysis of the raw water samples collected from the identified sampling points. Sampling was conducted from July 2011 to June 2018 (7 years). The collected water samples were analysed on monthly basis using advanced and state-of-art analytical equipments. Three Hundred and thirty six (336) water samples were analysed for pH, conductivity, chloride, sodium, potassium, hardness, alkalinity, precipitation potential, turbidity, colour, *E. coli*, organic carbon, chlorophyll, nitrite, ammonia, nitrates, phosphate, iron, manganese, and sulphate.			
Data format	Raw and analysed			
Parameters for data collection	To analyse the levels of various physicochemical and microbial parameters using standard methods. The levels of pH, conductivity, chloride, sodium, potassium, hardness, alkalinity, precipitation potential, turbidity, colour, total coliform, *E. coli*, total organic carbon, chlorophyll, nitrites, ammonia, nitrates, phosphate, calcium, magnesium, iron, manganese, and sulphate depicts the degree of raw water contamination and its suitability for human consumption.			
Description of data collection	The raw water quality data for Vaalkop, Klipdrift, Wallmansthal, and Cullinan water treatment plants (WTPs) were collected using standard methods. The principal aim of data collection was to assess the degree of contamination and determine the treatability index. The data on the levels of physical, chemical, and microbiological parameters were determined using the state-of-art analytical instruments and accredited standard methods.			
Data source location	Water treatment plants (WTPs) are placed in different location. Magalies Water has four water treatment plants, namely Vaalkop, Klipdrift, Wallmansthal, and Cullinan. Their localities are as follow:			
	Institution	Region	Country	WTP coordinates/ Localities
	Vaalkop	North West	South Africa	25°18′28.26”S,. 27°29′28.26”E
	Klipdrift	Tshwane	South Africa	25°22′59.36”S,. 28°18′34.99”E
	Wallmansthal	Tshwane	South Africa	25°34′34.22”S,. 28°19′40.66”E
	Cullinan	Tshwane	South Africa	25°40′30.81”S,. 28°31′45.78”E
Data accessibility	Data are included in this article and supplemented excel file.			

## Value of the Data

• The data presented is used to calculate the treatability index of raw water. This helps in the assessment of the degree of contamination for surface water and its treatment demands. Worryingly, the consumption of surface water contaminated by various forms of pollution can cause devastating effects to the health of the communities and different water end-users. As such, water quality assessment and treatability index evaluation helps in taking necessary steps to avoid using contaminated water and protect the health of the general public [1].
• Water quality specialists, engineers, environmentalists, scientists, end-users, citizens, developers, modellers, and planners will enormously benefits from this data. This will increase the knowledge of water quality in the identified research spots hence enhancing the understanding of the physicochemical and microbial properties of the surface water systems. As such, this data will play a key role during planning and modelling. It will also aid the custodians and interested parties to put mitigation measures to manage raw water resources.
• Due to limited published studies and up-to-date data on surface water quality and treatability index around Vaalkop, Klipdrift, Wallmansthal, and Cullinan WTP water catchment. The data will be useful in taking suitable measures for the government, planners, modellers, and other policy makers in supplying safe drinking water to different end-users.
• This data will also aid in giving a synoptic view of the variations in the raw water quality over a period of 7 years thus enabling the water treatment entities to identify emerging problems of concern and track-map their sources.
• The data deduced treatability index and trend analysis helps water treatment entities to understand historic trends, and this allows for easy projections, disaster preparedness, and planning.

## 1 Data Description

The physicochemical and microbial quality parameters of raw water were determined using standard methods. Data on pH, conductivity, chloride, sodium, potassium, hardness, alkalinity, precipitation potential, turbidity, colour, total coliform, *E. coli*, total organic carbon, chlorophyll, nitrites, ammonia, nitrates, phosphate, calcium, magnesium, iron, manganese, and sulphate were collected and reported. Close to 336 samples were collected over a period of 7 years and analysed using standard methods and procedures [2].

The data on statistical analysis of the physicochemical and microbial characteristics of raw water in Vaalkop water treatment plant (WTP) (from July 2011 to June 2018) are shown in Table 1 and 2. As depicted by the obtained data, most of the parameters were within the specified limits as stipulated in the South African National Standard (SANS) 241 for drinking water, except for turbidity, colour, total coliform, and *E. coli*, which were observed to be above the limits.

**Table 1:** Data on statistical analysis of the physicochemical characteristics of raw water in Vaalkop WTP.

Parameter	pH	Conductivity	Chloride	Sodium	Potassium	Hardness	Alkalinity, Tot.	Prec. Potential*	Turbidity	Colour
Units	-	mS/m	mg/L	mg/L	mg/L	mg/L CaCO_3_	mg/L CaCO_3_	mg/L CaCO_3_	NTU	mg/L Pt.
Min	7.0	24.5	13.0	0.0	3.8	65.0	16.0	-50.0	2.9	18.5
Max	9.0	161.5	97.0	87.5	12.8	249.3	329.1	22.2	234.8	1524.0
SDV	0.4	15.9	20.1	15.9	1.8	38.6	34.6	12.3	45.2	237.4
Aver.	8.2	62.2	70.5	50.5	7.2	172.9	120.2	2.7	24.9	107.2
Limits	9.7	170.0	300.0	200.0	50.0	300.0	250.0	20.0	5.0	15.0
Treatability	0.8	0.4	0.2	0.3	0.1	0.6	0.5	0.1	5.0	7.1

**Table 2:** Data on statistical analysis of the physicochemical and microbial characteristics of raw water in Vaalkop WTP.

Parameter	Total coliform	*E. coli*	Organic Carbon	Chlorophyll	Nitrites	Ammonium	Nitrates	Phosphate	Iron	Manganese	Sulphate
Units	Counts/100 mL	Counts/100 mL	mg/L	µg/L	mg/L	mg/L	mg/L	mg/L	mg/L	mg/L	mg/L
Min	95.6	0.0	1.9	0.9	0.0	0.0	0.2	0.0	4.4	2.4	20.0
Max	151925	825.0	15.0	185.0	34.8	2.1	7.4	51.0	2940.0	247.7	115.3
SDV	20102.6	107.5	2.5	38.1	3.9	0.4	1.4	5.5	429.2	46.3	18.3
Aver.	9894.8	35.6	7.2	35.4	0.9	0.2	1.4	0.7	200.3	44.9	73.2
Limits	10.0	0.0	10.0	100.0	0.9	1.5	11.0	10.0	2000.0	400.0	500.0
Treatability	989.5	3564.9	0.7	0.4	1.0	0.1	0.1	0.1	0.1	0.1	0.1

The average data and statistical analysis of the physicochemical and microbial characteristics of raw water in Klipdrift WPT are shown in Table 3 and 4. As shown in the obtained data, the analysed parameters were within the specified limits as stipulated in SANS 241 specifications for drinking water, except for turbidity, colour, total coliform, *E. coli*, organic carbon and nitrites, which were observed to be above the limits.

**Table 3:** Data on statistical analysis of the physicochemical characteristics of raw water in Klipdrift water treatment plant.

Parameter	pH	Conductivity	Chloride	Sodium	Potassium	Hardness	Alkalinity, Tot.	Prec. Potential*	Turbidity	Colour
Units	-	mS/m	mg/L	mg/L	mg/L	mg/L CaCO_3_	mg/L CaCO_3_	mg/L CaCO_3_	NTU	mg/L Pt.
Min	7.0	28.7	14.0	0.0	0.0	0.1	63.0	-52.0	0.9	-2.0
Max	9.4	58.1	59.7	52.0	12.2	168.6	206.3	30.9	30.3	169.0
SDV	0.5	5.8	9.3	9.9	2.2	29.4	23.4	11.5	6.2	24.6
Aver.	8.4	45.9	38.6	34.2	7.1	130.9	124.5	7.9	7.4	35.8
Limits	9.7	170.0	300.0	200.0	50.0	300.0	250.0	20.0	5.0	15.0
Treatability	0.9	0.3	0.1	0.2	0.1	0.4	0.5	0.4	1.5	2.4

**Table 4:** Data on statistical analysis of the physicochemical and microbial characteristics of raw water in Klipdrift WTP.

Parameter	Total coliform	*E. coli*	Organic Carbon	Chlorophyll	Nitrites	Ammonium	Nitrates	Phosphate	Iron	Manganese	Sulphate
Units	Counts/100 mL	Counts/100 mL	mg/L	µg/L	mg/L	mg/L	mg/L	mg/L	mg/L	mg/L	mg/L
Min	1608.0	11.0	1.0	0.0	0.0	0.0	1.4	0.0	0.0	13.8	1.6
Max	126325.0	8025.0	284.8	81.6	30.0	5.1	486.4	3.3	51.3	43.0	59.0
SDV	19312.2	1158.7	30.4	16.6	5.8	0.7	52.6	0.6	7.1	4.9	9.4
Aver.	17555.3	540.4	12.3	15.9	2.5	0.4	11.0	0.7	17.1	26.8	37.8
Limits	10.0	0.0	10.0	100.0	0.9	1.5	11.0	10.0	100.0	300.0	500.0
Treatability	1755.5	54041.9	1.2	0.2	2.8	0.3	1.0	0.1	0.2	0.1	0.1

The average data and statistical analysis of the physicochemical and microbial characteristics of raw water in Wallmansthal WTP are shown in Table 5 and 6. The obtained data meticulously depict high compliance of different water quality indicators to SANS 241 specifications, except for turbidity, colour, total coliform, *E. coli*, organic carbon, ammonia, and nitrites, which were observed to be above the specified limits.

**Table 5:** Data on statistical analysis of the physicochemical characteristics of raw water in Wallmansthal WTP.

Parameter	pH	Conductivity	Chloride	Sodium	Potassium	Hardness	Alkalinity, Tot.	Prec. Potential*	Turbidity	Colour
Units	-	mS/m	mg/L	mg/L	mg/L	mg/L CaCO_3_	mg/L CaCO_3_	mg/L CaCO_3_	NTU	mg/L Pt.
Min	2.4	29.2	9.0	14.0	3.8	67.0	69.5	-40.0	0.7	13.0
Max	9.7	109.1	62.8	93.0	12.4	185.0	190.4	30.0	27.4	147.0
SDV	0.8	9.0	9.3	10.2	2.0	20.9	23.1	9.4	5.8	20.2
Aver.	8.1	47.8	39.3	38.0	7.5	136.0	130.6	4.0	6.8	33.0
Limits	9.7	170.0	300.0	200.0	50.0	300.0	250.0	20.0	5.0	15.0
Treatability	0.8	0.3	0.1	0.2	0.2	0.5	0.5	0.2	1.4	2.2

**Table 6:** Data on statistical analysis of the physicochemical and microbial characteristics of raw water in Wallmansthal WTP.

Parameter	Total coliform	*E. coli*	Organic Carbon	Chlorophyll	Nitrites	Ammonium	Nitrates	Phosphate	Iron	Manganese	Sulphate
Units	Counts/100 mL	Counts/100 mL	mg/L	µg/L	mg/L	mg/L	mg/L	mg/L	mg/L	mg/L	mg/L
Min	520.0	2.0	2.5	-0.1	0.0	0.1	0.9	0.0	0.2	2.2	1.2
Max	83994.0	1318.0	21.0	79.0	50.9	55.3	17.9	3.1	23.7	37.4	139.5
SDV	17058.0	213.6	3.6	17.1	9.2	6.2	3.5	0.7	3.5	5.5	14.2
Aver.	14904.3	164.0	10.5	16.3	3.7	1.9	4.9	0.8	16.0	26.3	38.7
Limits	10.0	0.0	10.0	100.0	0.9	1.5	11.0	10.0	100.0	300.0	500.0
Treatability	1490.4	16401.5	1.1	0.2	4.1	1.2	0.4	0.1	0.2	0.1	0.1

The average data and statistical analysis of the physicochemical and microbial characteristics of raw water in Cullinan WTP are shown in Table 7 and 8. The obtained data was observed to conform to SANS 241 specifications for drinking water except for turbidity, colour, nitrites, total coliform, and *E. coli*, which were above the limit, however, total organic carbon, and ammonia were observed to be in the margin of the treatability index.

**Table 7:** Data on statistical analysis of the physicochemical and microbial characteristics of raw water in Cullinan WTP.

Parameters	pH	Conductivity	Chloride	Sodium	Potassium	Hardness	Alkalinity, Tot.	Prec. Potential*	Turbidity	Colour
Units	-	mS/m	mg/L	mg/L	mg/L	mg/L CaCO_3_	mg/L CaCO_3_	mg/L CaCO_3_	NTU	mg/L Pt.
Min	6.2	0.2	4.4	9.0	1.6	59.4	59.9	-154.9	6.2	23.6
Max	8.0	49.4	75.4	73.0	15.3	292.6	208.0	2.4	165.0	960.0
SDV	0.4	7.3	9.8	10.5	3.2	35.4	27.1	35.1	28.0	142.2
Aver.	7.2	31.7	20.2	22.4	6.8	111.6	101.5	-30.4	44.3	137.2
Limits	9.7	170.0	300.0	200.0	50.0	300.0	250.0	20.0	5.0	15.0
Treatability	0.7	0.2	0.1	0.1	0.1	0.4	0.4	-1.5	8.9	9.1

**Table 8:** Data on statistical analysis of the physicochemical and microbial characteristics of raw water in Cullinan WTP.

Parameters	Total coliform	*E. coli*	Organic Carbon	Chlorophyll	Nitrites	Ammonium	Nitrates	Phosphate	Iron	Manganese	Calcium	Sulphate
Units	Counts/100 mL	Counts/100 mL	mg/L	µg/L	mg/L	mg/L	mg/L	mg/L	mg/L	mg/L		
Min	3.0	0.4	3.0	0.4	0.0	0.0	0.1	0.0	6.3	7.0	2.0	0.7
Max	54500.0	2056.0	16.2	85.0	50.8	2.5	6.6	3.8	2280.0	9054.0	50.0	89.9
SDV	12526.8	312.6	2.5	15.4	5.6	0.5	1.4	0.5	459.8	1163.4	7.2	15.2
Aver.	9272.8	136.1	9.0	18.3	1.1	0.5	1.4	0.3	446.2	327.5	19.7	34.9
Limits	10.0	0.0	10.0	100.0	0.9	1.5	11.0	10.0	2000.0	400.0	300.0	500.0
Treatability	927.3	13611.7	0.9	0.2	1.2	0.3	0.1	0.0	0.2	0.8	0.1	0.1

## 2 Experimental Design, Materials and Methods

### 2.1 Study area

**Fig. 1:**
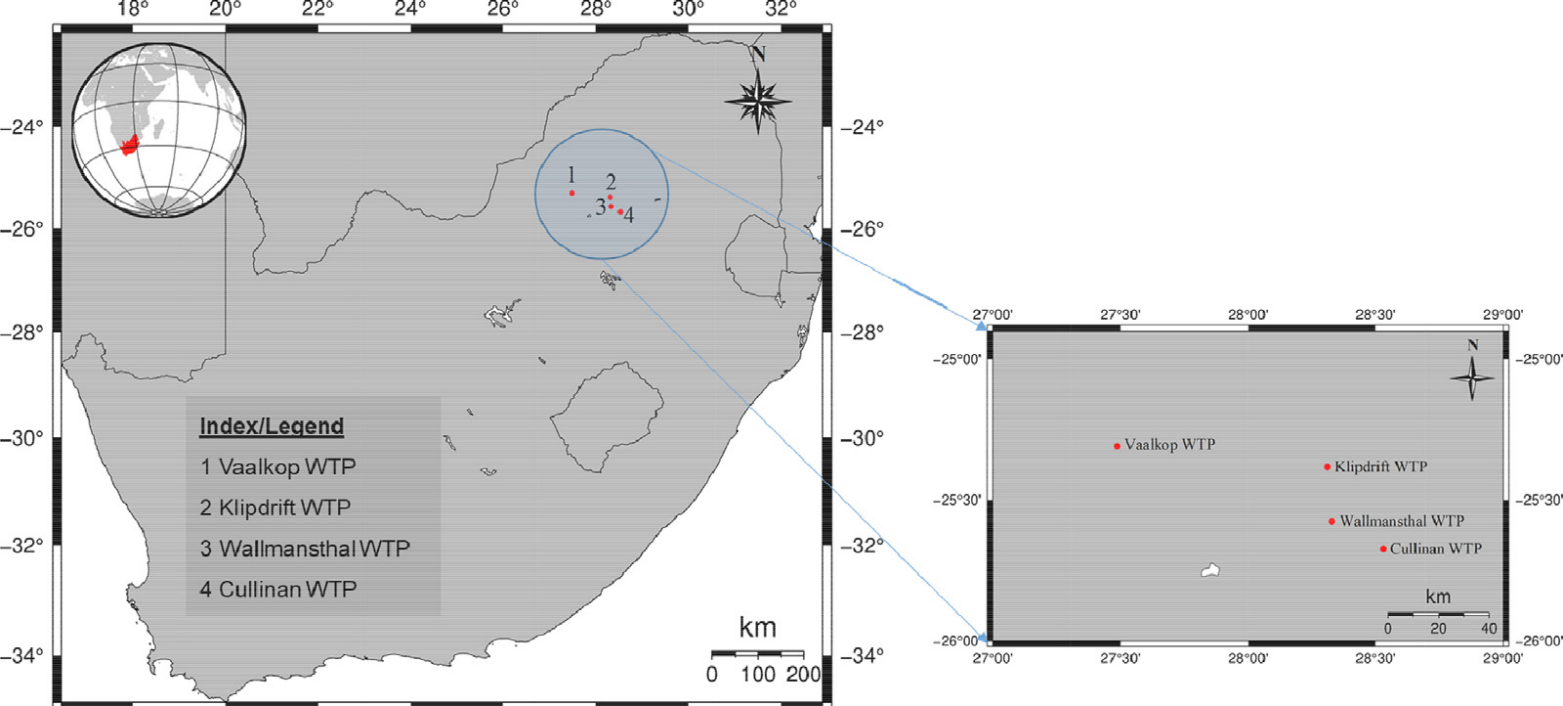
The map for the study area.

The study area is located in North West and Gauteng provinces, South Africa. The area of clean water distribution covers around 42 000 km^2^ and it footprint overlaps over three provinces (North West, Gauteng and Limpopo provinces). The map for the study area is shown in Fig. 1.

### 2.2 Sample collection and analyses

Raw water samples were collected at identified localities in Vaalkop, Klipdrift, Wallmansthal, and Cullinan water treatment plants (WTPs) (Fig. 1). Standard protocols and procedures for sampling were considered. After collection, the samples were immediately transported to the laboratory for analysis [1, 3]. The samples were then analyzed within 24 hrs from the period of collection. All water samples were analyzed according to the standard methods for examination of water samples [1, [3], [4], [5], [6]]. Quality control procedures were also observed throughout the sampling and analysis process.

### 2.3 Analysis of physicochemical and microbial parameters

State-of-art analytical instruments were used to determine physical, chemical, and microbial parameters of raw water. These equipments include: (i) inductively coupled plasma mass spectrometry (ICP-MS), XSeries 2, ICP-MS, supplied by Thermo scientific, from Hanna-Kunath-Str. 11 28199 Bremen, Germany. The ICP-MS was coupled to ASX-520 Auto sampler. (ii) Gallery plus photo spectrometer, Automated chemistry analyzer, Supplied by Thermo Fisher scientific, Made in Vantaa, Finland. (iii) HANNA Multi-parameter probe, HI-9828 Multi-Parameter Water Quality Portable Meter. Standard methods were also used to determine water quality determinants [1, 3, 5].

### 2.4 Data analysis, treatability index, and quality control

The physicochemical and microbial characteristics of the sampled raw water sources were used for statistical analysis. The data was analyzed using Microsoft office and SPSS. The analysed parameters include min, max, standard deviation (STDV) and average values. The analysis was solely based on 7 years of sampling from July 2011 to June 2018. The average data was benchmarked against the specified limits as stipulated in SANS 241 limits [2, 7, 8]. To effectively communicate the raw water quality, a robust treatability assessment tool was used. This tool is known as the treatability index (TI). The TI is defined as the ratio of the aliquots concentration and the maximum allowed limit, as shown in eq. (1):

(1) $$\text{Treatability}\;\text{index}=\frac{\text{Concentration}\;\text{of}\;\text{the}\;\text{aqueous}\;\text{parameter}}{\text{Maximum}\;\text{allowed}\;\text{limit}}$$

Where, the concentration of the parameter is the level of the contaminants in aqueous solution or the analyzed sample (mg/L, µg/L, etc.) and the maximum allowed limit is the specified limit from different water quality guidelines, standards and specifications such as SANS 241, WHO and EPA [9], [10], [11],. On that note, eq. (1) suggests that:

• When the treatability index is ≥1 the water will require treatment to comply to the required limits.
• When the treatability index is =1 the water will require limited to no treated to comply to the required limit.
• When the treatability index is ≤1 the water will require zero treatment to comply to the required limit.

## Declaration of Competing Interest

The author declare no known competing financial interests or personal relationships, which have, or could be perceived to have, influenced the work reported in this data article.

## Appendix Supplementary materials

Image, application 1

Image, application 2
